# Insulin Glulisine in Pregnancy – Experience from Clinical Trials and Post-marketing Surveillance

**DOI:** 10.17925/EE.2015.11.01.17

**Published:** 2015-04-11

**Authors:** Zoran Doder, Demi Vanechanos, Manfred Oster, Wolfgang Landgraf, Stephen Lin

**Affiliations:** 1. Global Pharmacovigilance & Epidemiology, Sanofi, Bridgewater, New Jersey, US; 2. Medical Affairs, Diabetes Division, Sanofi-Aventis, Frankfurt, Germany

**Keywords:** Diabetes mellitus, glulisine (Apidra®), congenital malformations, intra-uterine deaths

## Abstract

Pregnancies complicated by gestational diabetes or pre-existing type 1 or type 2 diabetes mellitus are associated with a higher rate of adverse outcomes compared with pregnancies in the background population. These outcomes include miscarriage, pre-term delivery, pre-eclampsia, perinatal mortality and congenital malformations. Insulin glulisine (Apidra^®^, Sanofi) is a rapid-acting insulin analogue indicated for the treatment of adults, adolescents and children 6 years or older with diabetes mellitus where treatment with insulin is required. Here, all post-marketing and clinical trials safety data with insulin glulisine in pregnancy available to Sanofi up to June 2014 are summarised together with the findings of a comprehensive literature search. Cumulatively, a total of 303 pregnancy exposures to insulin glulisine were received. Of these 303 pregnancy exposures, there were 116 live births, 12 spontaneous abortions, two late foetal intra-uterine deaths (>28 weeks), three elective abortions and 170 cases without a known pregnancy outcome. There were six cases of congenital malformations; of these, there were five live births; in the other case a live birth was not confirmed. The congenital malformations reported to date do not reveal a pattern of defects. In conclusion, the evidence to date does not suggest a causal association between insulin glulisine and an increased risk of pregnancy complications or congenital malformations.

## Diabetes in Pregnancy

It has been estimated that type 1 (T1D) and type 2 (T2D) diabetes mellitus (DM) affect at least 1 % of pregnancies.^1^ Gestational diabetes, which is characterised by glucose intolerance of variable severity that begins or is first diagnosed during pregnancy, may affect up to 15 % of all pregnancies worldwide.^2^ The outcomes differ, based on exposure to hyperglycaemia in the preconception period and in the first trimester, between gestational diabetes and T1D and T2D; therefore, different treatment regimens are indicated.

With the increasing prevalence of diabetes,^3^ the management of pregnancies complicated by diabetes is likely to become an increasingly common challenge in obstetric practice in many regions of the world.^4^ Pregnancy affects the maternal and foetal metabolism and exerts a diabetogenic effect even in women without diabetes.^5^ Pregnancy-specific hormones, such as human placental lactogen and prolactin, increase insulin resistance, demanding greater insulin production to achieve homeostasis of blood glucose levels in pregnancy.^6^ In pregnancy that is complicated by T1D or T2D, however, this demand for increased glucose production is not met and this leads to serious risks for both the mother and foetus.^7^ These include miscarriage, pre-term delivery, pre-eclampsia, perinatal mortality, macrosomia and congenital malformations.^4,8–11^

A dose-response association has been reported, i.e. a greater risk of congenital defects is linked to poorer periconceptional blood glucose.^12^ Maternal complications include hypertension, retinopathy and nephropathy and an increased risk of acute metabolic decompensation, such as hypoglycaemia and ketoacidosis.^13^ Excessive growth of the foetus may increase the risk of obstructed labour, shoulder dystocia, brachial plexus injury and the requirement for an instrumental delivery or Caesarean section (C/S).^13^ In the post-natal period, the babies of a mother with diabetes are at an increased risk of hypoglycaemia, respiratory distress and jaundice and are more likely to be admitted to a neonatal unit. Long-term, the children are at increased risk of diabetes themselves.

In a retrospective cohort study of 3,157 deliveries, compared with women without diabetes, those with pre-existing T1D or T2D were significantly older, of higher parity and they had more previous miscarriages.^4^ Women with pre-existing DM were more likely to deliver by emergency C/S (24.1 % versus 13.8 % in women without diabetes, odds ratio [OR] 2.67, 95 % confidence interval (CI) [1.63–4.32]; p<0.001), or elective C/S, (OR 6.73, 95 % CI [3.99–11.31]; p<0.001). The babies of the mothers with preexisting DM were significantly heavier, p<0.001; and more frequently macrosomic compared with women without diabetes (11.2 % versus 3.1 % in women without diabetes, OR 3.97, 95 % CI [2.03–7.65]; p=0.002).

**Table 1:** Unique Reports from All Sources of Insulin Glulisine (Apidra®)-exposed Pregnancies (Cumulative Cases to 30 June 2014)

	Cumulative Number
Totals reports of glulisine-exposed pregnancies	303
Reports from sponsored studies	23
Reports from literature	3
Reports from spontaneous sources	277

Strong evidence exists indicating that better outcomes for mother and child are achieved with preconception counselling and optimised diabetes management.^7,14^ Folate supplementation can reduce the incidence of foetal neural tube defects, which occur with greater frequency in foetuses of mothers with diabetes.^15–17^ Glucose control should be managed more vigorously in pregnancy;^18^ in addition to dietary changes, this requires more frequent monitoring of blood glucose levels and may involve additional injections of insulin or conversion to an insulin pump.^19^ Tightening of glucose control during the critical period of organogenesis reduces other congenital anomalies including sacral agenesis, caudal dysplasia, renal agenesis and ventricular septal defect.^20^

## Insulin Glulisine

Insulin glulisine (Apidra^®^) is a rapid-acting insulin analogue indicated for the treatment of adults, adolescents and children 6 years or older with DM where treatment with insulin is required. Manufactured by Sanofi using recombinant DNA technology, insulin glulisine differs from human insulin in that the amino acid asparagine at position B3 is replaced by lysine and the lysine in position B29 is replaced by glutamic acid.^21^ Insulin glulisine has been shown to be equipotent to regular human insulin.^22^ After subcutaneous administration, it has a more rapid onset and a shorter duration of action than regular human insulin.^22^

Insulin glulisine should be given shortly before (0–15 minutes) or soon after meals. Insulin glulisine should be used in regimens that include a longer-acting insulin or basal insulin analogue, and can be used with oral hypoglycaemic agents.^22^ It was first approved for marketing on 16 April 2004 in the US and is currently marketed in more than 100 countries worldwide. Based on prescription data and assuming an average dose of insulin glulisine is approximately 30 units/day the postmarketing cumulative exposure to insulin glulisine is estimated to be more than 4.1 million patient–years up to June 2014.

A recent literature review found no published data on the use of insulin glulisine in pregnancy.^13^ To provide the most up-to-date data available on insulin glulisine use in pregnancy, we analysed and summarised the experience with insulin glulisine in pregnancy from different sources, such as the Sanofi pharmacovigilance and clinical trials databases combined with the findings of an updated, comprehensive literature search.

## Methods

### Global Pharmacovigilance Database Pregnancy Exposure Reports

A cumulative summary of pregnancy exposure reports collected since the clinical development of insulin glulisine up to 30 June 2014 by Sanofi is included in this review. These reports include data collected from clinical trials conducted by the marketing authorisation holder Sanofi, as well as ‘spontaneous’ reports from consumers or healthcare professionals. The reports are categorised as follows:

- Prospective reports: pregnancies ascertained before the foetal outcome was known (i.e. live birth, malformation and abortion).
- Retrospective reports: those after the foetal outcome was known.

As is true for spontaneous reporting systems, congenital malformations that may have occurred but were not reported to Sanofi by the treating physicians could not be accounted for in this analysis.

### Literature Review

We conducted a comprehensive literature search in PubMed/MEDLINE (01 January 2001-11 September 2014) and Embase (01 January 2001–11 September 2014) for the search terms insulin glulisine AND pregnancy and insulin glulisine AND congenital malformation. The search results were independently assessed by all authors and full agreement between authors on the results of the literature search was achieved.

## Results

### Insulin Glulisine-exposed Pregnancies

Our literature search yielded 92 published papers, three of which reported insulin glulisine-exposed pregnancies.^23–25^ In addition, we identified and reviewed a total of 39 clinical trials conducted by the manufacturer in adult diabetes patients in which insulin glulisine was administered. From these trials, there were 23 unique reports of insulin glulisine-exposed pregnancies (cumulative total number of patients from clinical trials was 12,182, of whom 5,877 [48 %] were women). Another 277 spontaneous reports were available for assessment cumulatively to June 2014, resulting in 303 pregnancy exposures overall from all sources (see *Tables 1* and *2*).

As of 30 June 2014, a total of 303 pregnancy exposures have been documented from all sources, of which the outcome is known for 133 exposures (44 %). Nearly half (42 %) of exposures originated from Europe, 24 % from Japan, 9 % from the US and 25 % from the rest of the world.

### Congenital Abnormalities

Cumulatively, through 30 June 2014, six individual case safety reports of congenital anomalies (five live births; one unconfirmed live birth) were identified in the Sanofi pharmacovigilance database. Case reports included (1) truncus arteriosus and pulmonary hypertensive crisis; (2) oesophageal atresia; (3) atrial septal defect; (4) trisomy 21, pleural effusion, a cardiac septal defect and inferior vena cava dilatation; (5) cardiac disorder; and (6) talipes.

### Cases of Late Intrauterine Death

Two individual case safety reports of late intrauterine death were identified. One patient (age not provided) had a history of nephropathy, retinopathy, rubella and diabetes treated with insulin glulisine for T1DM during pregnancy (the diabetes duration of the patient was not reported). The foetus died at 36 weeks of pregnancy.

The other case involved a 25-year-old female patient who was treated with insulin glulisine and insulin lispro concomitantly. Relevant medical history includes one previous pregnancy resulting in a healthy baby weighing 3.6 kg at birth. During this second pregnancy, the patient gained 30 kg and delivered a stillborn baby weighing 5 kg at the thirty-eighth week of pregnancy. The patient reported that her glucose level was in general well controlled during pregnancy, but one severe nocturnal hypoglycaemia was reported. She was not informed of any defects with the unborn child, other than the thyroid level being high and the baby weighed 5 kg. The patient was at 11 weeks gestation (at the time of this report) in her third pregnancy and has been treated with insulin glargine at bedtime, and insulin glulisine.

**Table 2:** Outcome Reports of 303 Pregnancy Exposures (Cumulative Cases to 30 June 2014)

Outcomes	Prospective Reports	Retrospective Reports	Total
Unknown outcomes	–	–	170 (56 %)
Known outcomes	51	82	133 (44 %)
Live Births	43	73	116
Normal	32	53	85
Pre-term births^a^	11	18	29
Congenital anomaly	1	4	5
Spontaneous abortions (< 20 weeks’ gestation)	5	5	10
Spontaneous abortions (weeks’ gestation unknown)	1	1	2
Late foetal death (≥ 28 weeks’ gestation)	2	0	2
Elective abortions	0	3	3

*^a^Three cases of pre-term births also have congenital anomalies*.

## Other Adverse Events Unrelated to Congenital Abnormalities

Of 85 normal live births, reported adverse events included blood glucose fluctuation (mother was diagnosed with colorectal cancer 7 weeks after delivery); four babies experienced hyperbilirubinemia (3) or jaundice (1); one baby required phototherapy (probably due to hyperbilirubinemia, but unconfirmed); one baby was reported as larger for dates; one baby was admitted to the neonatal intensive care unit for 2 weeks on nasal cannula (reason unknown); and one baby experienced eczema. Other events included two cases of hypoglycaemia and one case of transient tachypnoea; all of these babies recovered from these events.

Of 29 pre-term births, four babies experienced transient respiratory distress (three babies recovered and no outcome was reported in the other case). Other reports included blood glucose decrease and jaundice (1), blood glucose fluctuations (1) and low birth weight (1). All of these babies recovered. No outcome was reported in one case of hyperbilirubinemia and one case of hyaline membrane disease and growth restriction. In one case, on the fourth day after birth, the baby experienced tremor and was hospitalised. Blood tests (which included glucose level measurement), head ultrasound and head tomography showed normal results and no diagnosis was provided. In this case, the mother experienced poor glycaemic control (hypoglycaemia and hyperglycaemia) and hypertension.

## Maternal Adverse Events

There were three cases in which pre-mature delivery was due to preeclampsia experienced by the mother, one case in which the pre-mature delivery was due to hypertension and sickle cell anaemia experienced by the mother and in one case the mother had experienced two episodes of hypoglycaemic coma and a decision was made to perform a C/S at 36 weeks’ gestation.

## Live Births

Out of the 116 live births: 85 were normal live births with no significant medical events reported during pregnancy. Of these, no medical events were reported for 73 cases (86 %).

## Discussion

In this current analysis, a total of 303 pregnancy exposures to insulin glulisine in pregnancy have been received. Of these, there were 116 live births, 12 spontaneous abortions, two late foetal intra-uterine deaths (> 28 weeks), three elective abortions and 170 cases with an unknown pregnancy outcome.

Regarding the 170 cases with unknown outcome, one case reported a congenital anomaly (cardiac defect) of the foetus at 13 weeks’ gestation. The physician did not consider that it was due to insulin glulisine but rather due to the result of unbalanced glucose levels prior to the pregnancy. The mother did not plan to terminate the pregnancy.

There were six cases of congenital malformations; of these, there were five live births and one malformation with unconfirmed live birth. The congenital malformations reported to date do not reveal a pattern of defects.

There were 29 pre-term births, 23 of these lacked sufficient information for a comprehensive causality analysis, and in the remaining six cases, the mother’s underlying condition provided an alternative explanation for the events (pre-eclampsia in three cases; poor glycaemic control of the mother in two cases; hypertension and sickle cell anaemia of the mother in one case).

The two cases of late intra-uterine death are confounded by the patients’ underlying uncontrolled DM.

The available literature for rapid- and long-acting insulin analogues does not indicate an increased risk of adverse outcomes of pregnancy or on the health of the foetus/new-born child when those are used during pregnancy.^13,26–30^ Prior to this analysis, there was no public information available concerning the potential safety of insulin glulisine in pregnancy.^13^ A direct comparison between results published for other insulin analogues and the current data for insulin glulisine is not possible due to different sources from which the data were collected (study versus post-marketing) and different methodologies that were used for analysis.

Reported outcome of pregnancy exposure cases ascertained retrospectively differed from outcomes ascertained prospectively. Retrospective ascertainment is subject to reporting bias, and this should be considered when attempting to use such data to estimate increased risk of pre-term births, spontaneous abortions or congenital malformations. Owing to the voluntary nature of the ‘spontaneous’ reports, it is often not possible to collect additional details after an initial report, leading to incomplete data in a significant portion of reports, especially with regard to the outcome of the pregnancy.

A major limitation of this analysis is that the pharmacovigilance data reflect spontaneous reports and in no way reflects the overall experience of insulin glulisine in pregnancy since unreported cases are not captured. Another weakness of the analysis is the lack of a comparator group. Further, the analysis was exploratory in nature and was not specifically aimed to find or disprove a causal relationship between insulin glulisine and maternal and foetal outcomes. In this analysis, information provided to Sanofi can only be assumed to be accurate. In addition, data from pharmacovigilance and clinical trial databases are likely to be incomplete in terms of all the clinical characteristics of the women with diabetes. Despite these limitations, this analysis is the first report of insulin glulisine use in pregnancy. The product has been in use for over 10 years and total estimated exposure is more than 4.1 million patient–years. The specific exposure in pregnant women is not known, however. The lack of evidence indicating a causal association between insulin glulisine and congenital malformations, despite extensive use, indirectly supports its safe use during pregnancy.

## Conclusion

Based on a review of the Sanofi global pharmacovigilance database combined with a comprehensive literature review, the available evidence does not suggest a causal association between insulin glulisine and an increased risk of pregnancy complications or congenital malformations.
